# Genetic association of preeclampsia to von Willebrand factor and its size-regulator ADAMTS13

**DOI:** 10.21203/rs.3.rs-5685318/v1

**Published:** 2025-07-08

**Authors:** A. Inkeri Lokki, Michael Triebwasser, Emma Daly, Mitja I. Kurki, Markus Perola, Kirsi Auro, Anuja Java, Jane E. Salmon, Seppo Heinonen, Eero Kajantie, Juha Kere, Riitta Lassila, Mark Daly, John P. Atkinson, Hannele Laivuori, Seppo Meri

**Affiliations:** University of Helsinki; University of Michigan; Hospital and Harvard Medical School; University of Helsinki; National Institute for Health and Welfare; National Institute for Health and Welfare; Washington University School of Medicine; Weill Medical College of Cornell University; University of Helsinki and Helsinki University Hospital; Norwegian University of Health and Technology; Karolinska Institutet; University of Helsinki, Helsinki University Hospital; University of Helsinki; Washington University School of Medicine; Tampere University; University of Helsinki

**Keywords:** preeclampsia, pregnancy, coagulation cascade, von willebrand factor, ADAMTS13, genetic association

## Abstract

Preeclampsia is a common pregnancy-specific vascular disorder that develops during the second half of pregnancy. Preeclampsia shares features with thrombotic microangiopathies. Here we analyzed whether sequence variants in the coagulation system genes predispose to preeclampsia. We performed targeted exomic sequencing of 58 genes in a total of 615 preeclamptic women and 2094 controls. A common missense variant rs1800385 (Val1565Leu) in the gene coding for von Willebrand Factor (*VWF*) (OR=1.72, p-value=3.57E-4) and a low-frequency missense variant rs41314453 (Ala732Val) in the gene coding for a disintegrin and metalloproteinase with a thrombospondin type 1 motif, member 13 (ADAMTS13) (OR=1.97, p-value=0.044) were associated with preeclampsia. rs41314453 is known to decrease ADAMTS13 expression and activity. Thus, the reduced enzyme activity could promote the formation of large vWF polymers on endothelial cells and platelets and thereby increase vascular prothrombotic activity in preeclampsia. Our results support a role for an impaired ability of ADAMTS13 to limit VWF polymerization in the pathogenesis of PE. Ultralarge multimers of VWF could mediate platelet accumulation in the turbulent intervillous spaces in preeclamptic placentae, calling upon novel therapeutics to control the VWF-ADAMTS13 axis in severe cases having low ADAMTS13 in the presence of high VWF levels and multimerization.

## Introduction

Preeclampsia (PE) is a common pregnancy-specific vascular disorder with diverse clinical characteristics. It affects approximately 3% of pregnancies and accounts for over 50,000 maternal and 900,000 perinatal deaths annually^1^. No specific treatment, other than delivery, is available for PE. For prevention, low-dose aspirin administered from < 16 weeks of gestation has been suggested to reduce the risk of preterm PE (resulting in delivery before 37 wks of gestation) in women at high risk for PE, but its use remains controversial^2^. Despite common signs, proteinuria and hypertension, the etiology of PE could be heterogeneous, especially in a subset of cases.

There is a familial predisposition to PE and strong epidemiological evidence suggests that the risk for PE is inherited^3^. However, the individual variant effects of the candidate genes discovered thus far are modest. Finnish population presents an opportunity to study complex diseases, because the allele frequencies observed in the modern Finnish population result from several bottleneck events in the founder population which helps to identify relevant pathways for disease pathogenesis due to the enrichment of associating variants^4^.

The coagulation system is activated by changes in the vascular endothelium, and by platelet activation, adhesion, aggregation and interaction among leukocytes. In these processes, platelet interactions with von Willebrand factor (*VWF*) are an important contributor. Dysregulation of the platelet activity and coagulation system may result in thrombotic microangiopathies, including hemolytic anemia, and thrombocytopenia in women with PE^5^. No firm consensus regarding the role of platelets and coagulation biology in the development of PE has been reached^6–8^. Early results linking common variants such as FV Leiden and the prothrombin 3′ UTR variant to PE risk have not been replicated in larger studies^9^.

To investigate the role of the genetic burden of coagulation proteins in PE, we designed a targeted exome sequencing study to screen the exons and splicing sites of genes involved in blood coagulation and its regulation.

## Results

In the 58 selected genes we discovered 107 annotated variants and 151 presumably benign variants (data not shown). Key results of this association analysis are shown in Table 2 (significant and borderline significant variants noted). The significantly associated variants in Table 2 are all listed as variant of unknown significance by the American College of Medical Genetics (ACMG) classification^10^. Overall, the most significant associations were available in the hemostasis axis, including VWF and its size-regulating ADAMTS13 enzyme. Also, two protective antithrombin variants were discovered.

**Figure 1:**
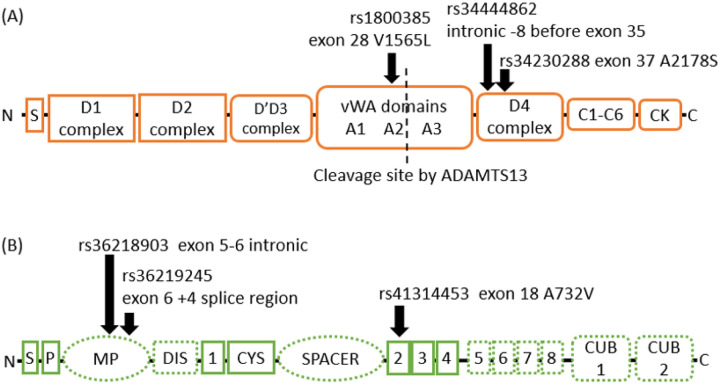
Domain structure of von Willebrand factor and ADAMTS13 with associating variants indicated with black arrows. Panel (A) von Willebrand factor (VWF) protein consists of eight functional classes of domains (29 in total, not shown) encoded by 52 exons (not shown). The domains in the propeptide region consisting of 741 amino acids are marked with sharp-cornered boxes and the domains that produce the mature VWF consisting of 2050 amino acids are marked by round-cornered boxes. The cleavage site of ADAMTS13 in the A2 domain is indicated with a dashed vertical line. Panel (B). ADAMTS13 consists of 12 domains that are encoded by 29 exons. The domains of the ADAMTS13 are a signal peptide (S), a propeptide (P), a metalloprotease domain (MP), a disintegrin domain (DIS), 8 thrombospondin type 1 domains (1–8), a cysteine-rich region (CYS), a spacer domain and two CUB domains. The domains that bind VWF are marked with dashed outlines. The disintegrin and spacer domains cleave the VWF A2 domain. The thrombospondin type domains 5–8 and CUB domains bind D4 and CK domains of VWF.

The gene coding for von Willebrand Factor (*VWF*) wasassociated with PE by three likely or probable LoF variants with significant p-values (Figure 1A). Rs1800385 (p.Val1565Leu) in the middle of the *VWF* gene is a missense variant that increases the risk for PE (OR=1.72, 95% confidence interval (CI) 1.27–2.32, p-value=3.57E-4). The intronic variant rs34444862 located 8 base pairs downstream of exon 35, and the second missense variant rs34230288 (p.Ala2178Ser) increase the risk for PE (OR=2.30, 95%CI=1.19–4.35, p-value=0.01; OR=2.2, CI 1.11–4.17, p-value=0.017, respectively).

In *ADAMTS13*, the intronic variant rs36218903 increased the risk for PE (OR=3.06, CI 95%=1.42–6.53; p-value=0.002), while the splice region variant rs36219245 decreased risk (OR=0.57, 95% CI 0.40–0.80; p-value=8.62E-4), (Figure 1B). The missense variant rs41314453 (Ala732Val) in *ADAMTS13* had a suggested increase in PE risk (OR=1.97, 95% CI=0.99–3.78; p-value=0.044).

We also found that in our cohorts rs5878 and rs5877 in *SERPINC1* encoding for antithrombin (III), a critical plasma protease inhibitor and a member of the serpin superfamily, decreased the risk for PE (OR= 0.85 (95% CI=0.74 – 0.98), and 0.86 (95% CI=0.74–1), p-value=0.02 and 0.03, respectively).

**Figure 2:**
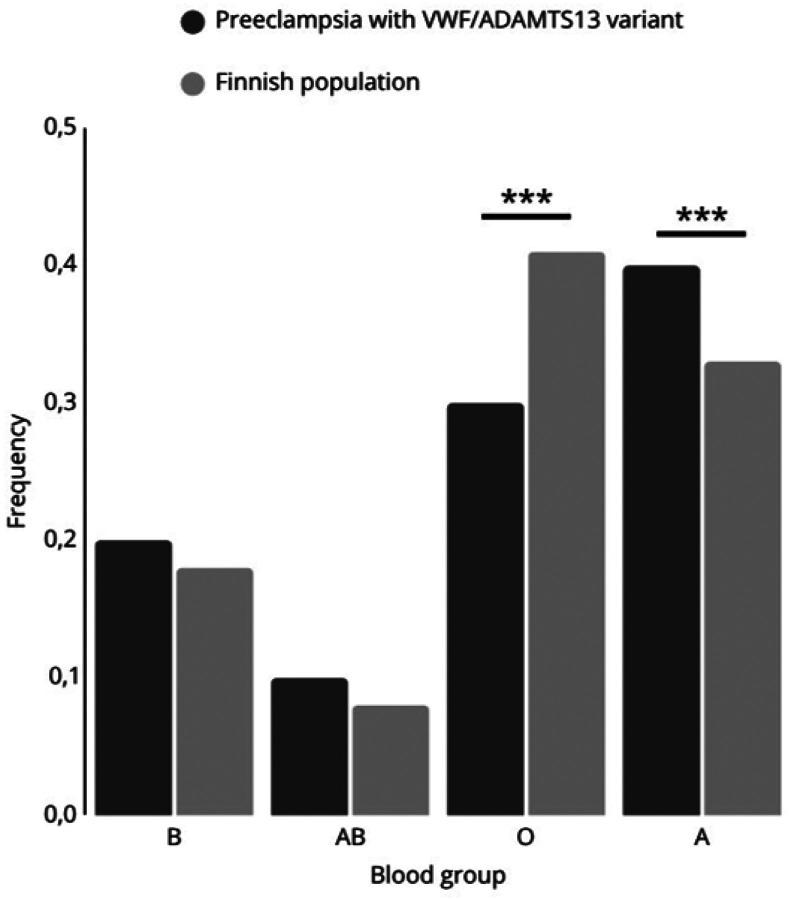
Frequency of blood groups of preeclampsia patients with any predisposing VWF/ADAMTS13 allele (pe; N=80) and in the Finnish population (fin; N=5536, source: Finnish Red Cross Blood Service). *** indicates p<0.0001.

Comparison of the frequency of blood groups between patients carrying *VWF* and/or *ADAMTS13* variants in the Finnish population revealed that women affected by PE blood group A are significantly more prevalent (X^2^ = 16.227, p<0.0001; Figure 2). In contrast, blood group O was significantly underrepresented in the PE patients (X^2^ = 21.403, p<0.0001).

## Discussion

Our results suggest that genetic variants in *VWF* and in its size and functional regulator ADAMTS13 associate with primary hemostasis abnormalities. Variants in genes coding for both proteins may predispose to PE. Women with blood group A are at particular risk of VWF-mediated PE, while mothers with blood group O have a lower incidence of PE.

These observations further support the proposed causative role of platelet dysregulation in a specific subgroup of PE. Importantly, we found associations within the VWF and ADAMTS13 axis, that cooperates to promote platelet-vascular wall interactions. A decrease in ADAMTS13 activity and an increase in VWF levels have also been previously associated with PE^11^, although the underlying and causative mechanisms in the VWF pathway have been under debate^12,13^. It is possible that VWF abnormalities are particularly associated with PE with severe features^14^. Common genetic variants within other coagulation genes are associated with PE^15^. Two of the discovered common PE-associating variants rs5878 and rs5877 in *SERPINC1* that encode antithrombin are related reduced generation of thrombin and formation of fibrin. While we were able to confirm the reported association between Factor V Leiden (rs6025) and PE, the literature provides sparse insight into the potential associations we discovered in other *F5* loci or variants discovered in *F2, F7*, and *SERPINA5*. Overall, the link between common coagulation variants, regulation of the coagulation system and an increased risk of preeclampsia^16,17^ was strongly corroborated by our data.

VWF is a plasma, platelet and endothelial glycoprotein that maintains hemostasis by generating multimers, which induce platelet aggregation and bind several proteins on activated endothelial cells in the vascular wall. Thereby, the multimers can lead to loss of vascular endothelial integrity^18^. This is particularly relevant in the placental vasculature due to its specific hemodynamic conditions. To prevent excessive platelet responses and coagulation, VWF oligomers emerging from endothelial cells or activated platelets are proteolytically cleaved by the ADAMTS13 enzyme^19,20^. ADAMTS13 cleaves VWF between tyrosine and methionine at position 842–843. Mutations in the *ADAMTS13* gene or, more commonly, autoantibodies against the ADAMTS13 enzyme cause thrombotic thrombocytopenic purpura (TTP).

**Figure 3:**
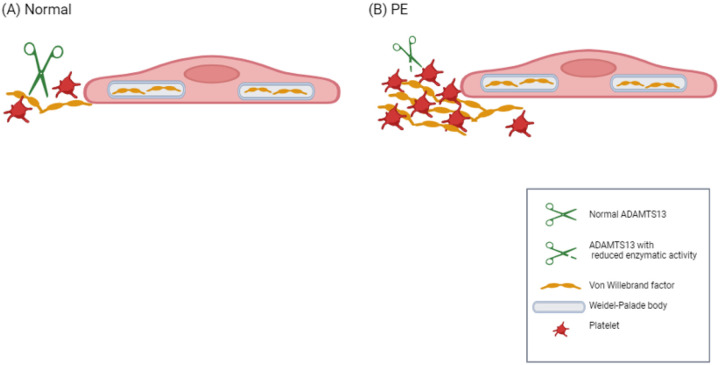
Schematic depiction of the proposed role of the ADAMTS13 Ala732Val variant in PE. In normal pregnancy (panel A), the cleavage of Weibel-Palade body-derived VWF from endothelial cells by ADAMTS13 prevents excessive VWF multimerization. In preeclampsia pregnancies (panel B) with rs41314453*T of *ADAMTS13*, the amount or enzymatic activity of ADAMTS13 is reduced thereby enhancing multimeric VWF and platelet aggregation. Created with BioRender.com.

The variant rs34230288 results in the replacement of the alanine at position 2178 with a serine in VWF. This variant has been observed in a patient who was *in cis* heterozygous for two *VWF* mutations and suffering from a noncanonical type 2B von Willebrand disease characterized by low VWF activity^21^. Similarly, the variant rs1800385 results in the replacement of valine at position 1565 by leucine resulting in significantly elevated ADAMTS13 activity but available data is insufficient to ascertain it’s functional relevance^22^. Rs41314453 is the main genetic determinant of ADAMTS13 activity. It is important to note that it is in linkage disequilibrium with several intronic variants in *ADAMTS13* and variants in the regulatory regions of neighbouring genes^23^. The total effect of rs41314453 is dependent on the sequence context, which may influence the extent and direction of its effect on gene expression^24^. It has been estimated that the variant reduces ADAMTS13 levels by approximately 40%^23,24^. Although this magnitude of a decrease does not reach levels that are considered significant in TTP (< 10%), it may be significant in the context of the strong triggers such as pregnancy. The product of the *ADAMTS13* gene with the minor allele T of rs41314453 has up to 29% less VWF cleavage activity than the protein coded by the gene with the major allele^24^. Thereby, rs41314453 may increase the risk for platelet deposition by accumulation of ultralarge VWF multimers (Fig. 3). In TTP, the accumulation of platelet-super-adhesive ultralarge VWF multimers on vascular endothelium leads to the spontaneous formation of microthrombi. Pregnancy is also one of the well-known triggers to precipitate attacks of TTP^5^.

We recorded the blood groups of the women due to their role in association with VWF levels. Persons with blood group O have 30% lower VWF expression than the other blood groups, and blood group O has implications for platelet physiology^25^. Previously, blood groups A and more convincingly AB have been linked to a modestly increased risk of PE^26,27^. In addition, blood group A has been found to predispose to severe COVID-19, while blood group O is protective against infection and microthrombosis^28^. Concurrently, blood group O carries a 30% lower level of VWF, which may be highly elevated in COVID-19^29,30^. COVID-19 infection is also an independent risk factor for PE^33^. Our observed genetic variants of VWF, ADAMTS13 and non-O blood groups, likely contribute to the pathogenesis of PE. Furthermore, these findings may be helpful in the future to risk stratify patients and target novel therapies based on the specific analysis of VWF and ADAMTS13 biomarkers^31^

The findings of our study may explain aspects of the pathophysiology of PE and clinical observations related to the preventive use of aspirin. In PE, the placental intervillous blood flow is perturbed due to the lack of vasodilation in the spiral arteries, and local high shear forces prevail, promoting platelet-VWF interactions^32,33^. This also increases the risk of local red blood cell lysis and promotes the release of ADP and thromboxane A2, which are known to further activate platelets. ADP increases the expression and release of VWF on platelets^34^. Associated activation of the complement system results in the formation of C5a and of membrane attack complexes, which can further activate platelets and induce release of VWF from endothelial cell Weibel-Palade bodies^35–37^. Subsequent reduced ability of ADAMTS13 to cleave VWF multimers would thus promote formation of platelet aggregates, which have been shown to be resistant to ADAMTS13^38^. On the other hand, VWF has been shown to protect the endothelium from complement-mediated injury^39^. Aspirin has been shown to reduce expression of VWF on platelet surfaces^34^. Thereby it may partially compensate for the procoagulant effect of rs41314453 on *ADAMTS13*. Reduced platelet activity may improve blood flow in the intervillous space and reduce local ischemia and the severity of PE. TTP is associated with adverse pregnancy outcomes including PE^40^. Previously, a patient suffering from TTP due to a mutation in *ADAMTS13* experienced a successful pregnancy under prophylactic treatment by aspirin^41^. More recently, novel drugs to influence the VWF-ADAMTS13 axis have emerged. Drugs like caplacizumab or recombinant ADAMTS13 could thus potentially be used in severe cases that are linked to high level of VWF multimerization and thrombosis^42^.

In TMA, activation of the coagulation cascade and complement systems often go hand in hand^43^. Similarly, pregnancy is an inflammatory and procoagulative state. In a blood proteomic study, the most different expression patterns between preeclamptic patients and controls were observed in complement and coagulation pathways and platelet function and VWF were also implicated^44^. Thereby patients with a predisposing complement and/or coagulation pathway variants may present with TMA-like PE^45^.

This study was limited by unavailability to study VWF and ADAMTS13 activity or their biomarkers. Furthermore, complete blood cell counts were not measured routinely thereby rendering the analysis of this data inconclusive. The effect of blood group for PE risk in carriers of *VWF* and *ADAMTS13* variants requires further investigation in other well-described case-control cohorts representing varied populations.

In summary, our findings demonstrate a link between PE and two important and related hemostatic components VWF and ADAMTS13. Our results support the concept that in some cases, PE with severe features may present as a thrombotic microangiopathy^45^. The fact that PE-associated rs41314453 reduces ADAMTS13 level suggests that the ADAMTS13-VWF axis and regulated VWF size or multimerization are important in preventing PE. Our results may also relate to aspirin, which may show preventive properties against preeclampsia in high-risk individuals. However, in the future based on laboratory assessment of VWF and ADMTS13, novel medications, such as caplacizumab and recombinant ADAMTS13 should be evaluated in PE.

## Methods

### Patient cohorts

Two independent case-control cohorts, The Finnish Genetics of preeclampsia Consortium (FINNPEC) cohort and the national FINRISK study cohort were investigated. The study rational is described in detail in the supplementary data. In the final association analyses, we included genotypes of FINNPEC and FINRISK population cohorts, leading to a combined total of 615 cases and 2094 controls. For FINNPEC, all women provided a written informed consent, and the FINNPEC study protocol was approved by the coordinating Ethics Committee of the Hospital District of Helsinki and Uusimaa. (FINRISK license 8/2016)^46^. The patients and controls from the FINNPEC cohort are characterized in the Supplementary table S1. The National FINRISK Study description and ethical approvals are available online: https://www.thl.fi/en/web/thlfi-en/research-and-expertwork/population-studies/the-national-finrisk-study. This study was conducted in accordance with the Declaration of Helsinki.

### Targeted Sequencing and Capture Enrichment

Libraries from genomic DNA were prepared in-house (Washington University School of Medicine)^47^. Enzymes were purchased from Enzymatics (Beverly, MA). Briefly, the ends of sheared genomic DNA fragments were repaired by treatment with T4 DNA Polymerase and T4 DNA Polynucleotide Kinase, which phosphorylates the 5’ hydroxyl. Next, an adenosine was added to the 3’ position at each end of the DNA fragment with Taq Polymerase. Illumina adapters with an overhanging “T” were ligated onto the DNA fragment followed by bead-based size selection to remove adapter-dimers and fragments below the desired size. A barcode consisting of a unique index sequence was added by PCR by targeting the two ligated universal adapters on each fragment end. Sequence capture hybridization and other laboratory methods are described in the Supplementary data. The studied genes and intronic loci of interest are listed in Table 1.

Fisher’s exact t-test was used as the primary test of association, and differences in frequencies of variants with p-value < 0.05 were considered significant. Significant and borderline significant variants are listed in Table 2. In addition to the statistical probability test, odds ratios (OR) with 95% confidence intervals (CI95) were calculated for all variants.

Comparison of the distribution of P values in benign (synonymous, intronic/intergenic; 151 observed variants) vs. annotated (missense, truncating, essential splice and splice region; 107 observed variants) variants indicate that the expected incidence of two annotated variants with p<0.001 is less than 0.01 in our data, compared with 0 observed variants with p<0.001. The lack of inflated P values indicates that confounders, such as stratification, are not causing false positives. Loss of function (LoF) analyses were done *in silico* for all genes with associating variants by the Loss of Function – tool of the Variant Effect Predictor (VEP) (https://github.com/ensembl-variation/VEP_plugins/blob/master/LoFtool.pm). In the LoF tool, the following annotations were calculated: LoF score < 0.2 indicates a probably damaging variant, LoF score 0.2–0.7 possibly damaging and LoF score < 0.7 a benign variant. 5/7 of the genes in Table 2 had scores < 0.2, suggesting a probable LoF.

Data were analyzed using PlinkSeq, Plink^48^ and R. Kaviar^49^. VEP Build 37 was used for additional annotations^50^.

## Supplementary Files

This is a list of supplementary files associated with this preprint. Click to download.

- supplementarydataclean.docx

## Figures and Tables

**Table 1.** Targeted coagulation-associated genes and intronic loci.

Coagulation-associated genes				Coagulation loci	
				Gene	SNP
*ABO*	*F7*	*KLKB1*	*SERPNA5*	*F2*	rs1799963
*ADAMTS13*	*F8*	*KNG1*	*SERPNB2*	*F5*	rs6020
*ADRA2A*	*F9*	*MRVI1*	*SERPNC1*	*SERPINE1*	rs2227631
*CD36*	*FGA*	*PEAR1*	*SERPND1*		
*F10*	*FGB*	*PIK3CG*	*SERPNE1*		
*F11*	*FGG*	*PLAT*	*SERPNE2*		
*F12*	*GP1BA*	*PLAU*	*SHH*		
*F13B*	*GP1BB*	*PLG*	*STX2*		
*F2*	*GP5*	*PROC*	*STXBP5*		
*F2R*	*GP6*	*PROCR*	*SVIL*		
*F2RL1*	*GP9*	*PROS1*	*TFPI*		
*F2RL2*	*IPCEF1*	*PROZ*	*TFPI2*		
*F2RL3*	*ITGA2B*	*SELP*	*VSGI4*		
*F3*	*ITGB3*	*SERPINA1*	*VWF*		
*F5*	*JMJD1C*	*SERPINA10*			

**Table 2.** Variants with significant or suggestive associations to preeclampsia within genes coding for coagulation proteins. Loss of function (LoF) is given per gene. All variants in Table 2 had a The American College of Medical Genetics and Genomics classification of variant of uncertain significance due to not enough evidence.

RSID number	Gene name	P-value	OR (95% confidence interval)	MAF_cases_	MAF_controls_	Consequence (distance from exon, base pairs)	LoFtool*
rs1800385	*VWF*	3.57E-4	1.72 (1.27 – 2.32)	0.059	0.035	missense variant, V1565L	0.03
rs34444862	*VWF*	0.01	2.30 (1.19 – 4.35)	0.015	0.007	intron variant (−8)	
rs34230288	*VWF*	0.02	2.18 (1.11 – 4.17)	0.014	0.007	missense variant, A2178S	
rs36219245	*ADAMTS13*	8.62E-4	0.57 (0.40 – 0.80)	0.053	0.089	splice region variant (+4)	0.52
rs36218903	*ADAMTS13*	2.28E-3	3.06 (1.42 – 6.52)	0.012	0.004	intron variant (−33)	
rs41314453	*ADAMTS13*	0.04	1.97 (0.99 – 3.78)	0.013	0.007	missense variant, A732V	
rs5898	*F2*	0.03	1.31 (1.01 – 1.69)	0.077	0.060	synonymous variant, P395P	0.13
rs2301515	*F5*	8.06E-3	1.21 (1.05 – 1.39)	0.014	0.007	intron variant (−50)	0.09
rs6023	*F5*	0.01	1.48 (1.07 – 2.03)	0.050	0.034	splice region variant (+7)	
rs6025	*F5*	0.05	1.49 (1.01 – 2.21)	0.030	0.021	missense variant, R534Q	
rs9332688	*F5*	0.02	2.10 (1.07 – 3.98)	0.014	0.007	intron variant (−32)	
rs900258823	*F5*	0.05	1.49 (1.01 – 2.21)	0.030	0.021	intron variant (−7098)	
rs6042	*F7*	0.02	1.32 (1.04 – 1.67)	0.086	0.067	synonymous variant, H176H	0.07
rs6109	*SERPINA5*	5.70E-3	1.22 (1.06 – 1.41)	0.313	0.272	intron variant (−34)	0.06
rs6115	*SERPINA5*	0.04	1.15 (1.01 – 1.32)	0.367	0.335	missense variant, S64N	
rs5878	*SERPINC1*	0.02	1.17 (1.02 – 1.35)	0.316	0.282	synonymous variant, Q337Q	na
rs5877	*SERPINC1*	0.03	1.17 (1.01–1.34)	0.301	0.269	synonymous variant, V327V	na

* LoF tool – (loss of function tool); score of LoF susceptibility per gene < 0.2 = probably damaging, 0.2–0.7 = possibly damaging, > 0.7 = benign

na – not available

RSID – Single nucleotide polymorphism identifier, OR – Odds Ratio, MAF – Minor Allele Frequency

## Data Availability

The datasets used and/or analysed during the current study available from Professor Hannele Laivuori on reasonable request.

## Data availability statement

The datasets generated during and/or analysed during the current study are available from Professor Hannele Laivuori on reasonable request.
